# Supplementary material for: miR-9a modulates maintenance and ageing of Drosophila germline stem cells by limiting N-cadherin expression
Source: Nat Commun. 2017 Sep 19;8:600. doi: 10.1038/s41467-017-00485-9 (PMC5605507; doi:10.1038/s41467-017-00485-9)
Supplement: Supplementary file 2 — Supplementary Information [file 41467_2017_485_MOESM2_ESM.pdf]

Title: Supplementary Information

Description: Supplementary Figures

Title: Peer Review File

Description:

Supplementary Information

Supplementary figures

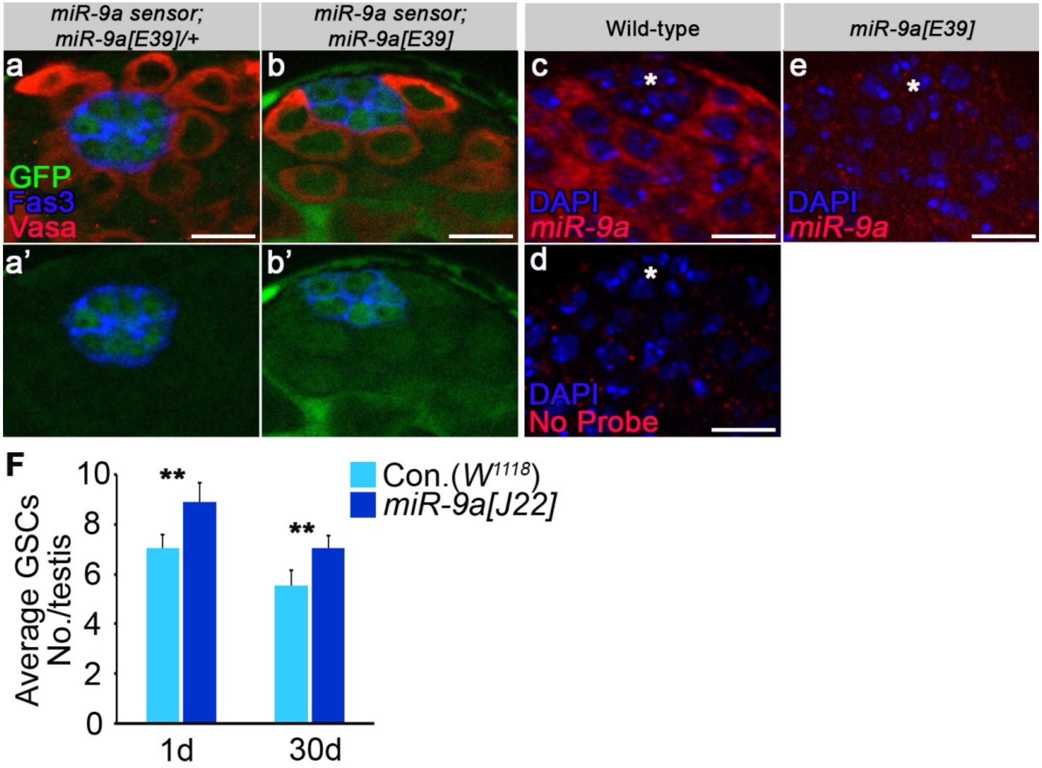

Supplementary Figure 1: *miR-9a* is expressed in GSCs and spermatogonia cells. (a-b') Representative images of testis immunostained for Vasa (red), Fas3 (blue) and detected for GFP (green). **(a,a')** Control: *miR-9a* GFP sensor expressed in heterozygous *miR-9a* mutants (*miR-9a sensor;miR9a[E39]*/+) shows GFP expression in the hub and lack of GFP in GSCs and spermatogonia. **(b,b')** *miR-9a* GFP sensor expresses GFP in GSCs and spermatogonia of *miR-9a* mutants (*miR-9a sensor;miR9a[E39]*). **(c-e)** FISH for *miR-9a* (red) and DAPI (blue) Asterisks mark the hub. **(c,d)** Testis of wild-type (w^{1118}) **(d)** FISH control without probe. **(e)** FISH control of testis from *miR9a[E39]* null mutants. Scale-bars, 10µm. **(f)** Shown are average number of GSCs per testis along with 95% confidence interval (error bars). The total number of testes scored: con. (w^{1118}) 1-day (*n*=26), 30-days (*n*=34); *miR-9a[J22]* 1-day (*n*=27), 30-days (*n*=24). Statistical significance was determined as in Fig. 1d. P values ** ≤ 0.005 in young and aged *miR-9a[J22]* compared to con. (w^{1118}).

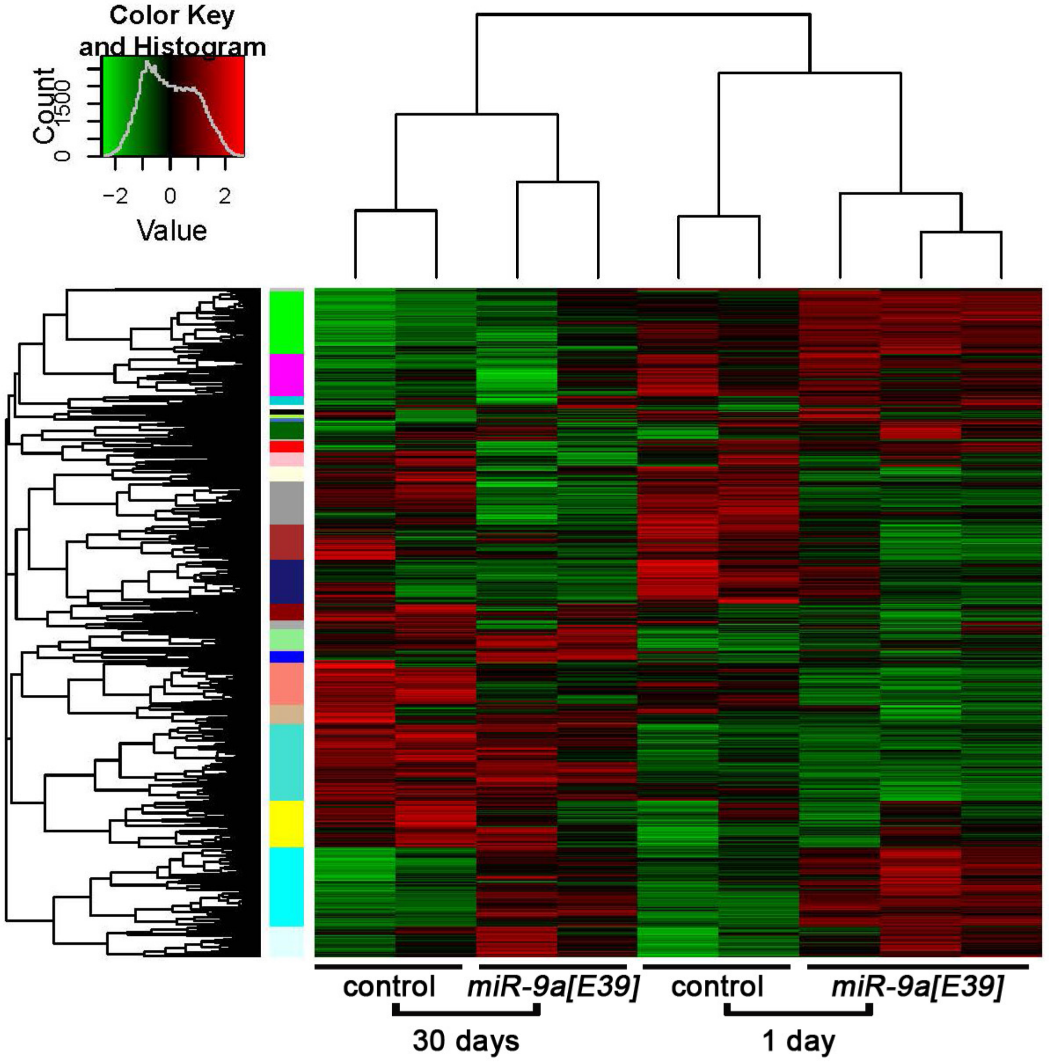

Supplementary Figure 2: Heatmap clustering of transcriptome analysis. Differentially expressed *Drosophila* genes from testes of: 1- and 30-days old control (*w*[1118]) and *miR-9a[E39]* mutants. The heatmap color values correspond to the z-scores of RNA-Seq CPM values; Horizontal axis: clustering by samples; Vertical axis: clustering by genes, where each color strip represents a clade of similarly expressed genes. Note clustering of biological repeats and age.

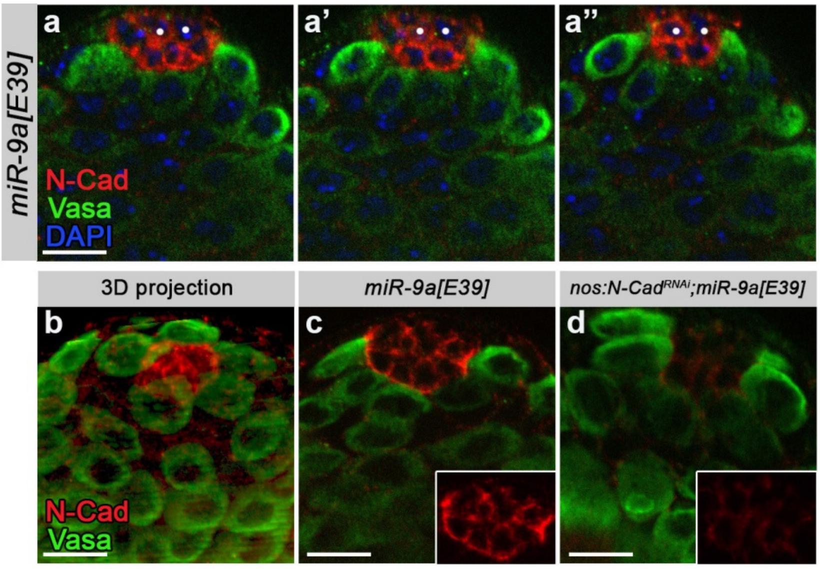

Supplementary Figure 3: Most hub cells form adherent junction with GSCs. (a-a'') Z-sections (1µm intervals) of *miR9a[E39]* niche stained with N-Cad (red), Vasa (green) and DAPI (blue; 3b image). White dots mark hub cells unconnected to GSCs present in section a, but connect the GSCs present in section a'' indicating that GSCs pile around the hub in multiple layers. **(b)** 3D projection of 10 Z-stacks showing the spherical structure of the GSCs niche (7 GSCs). **(c-d)** Representative images of testes taken at the same exposure time from *miR-9a[E39]* mutants (b, n=20) and from N-Cad^{RNAi} in GSCs and progenitor germ cells of *miR-9a[E39]* mutants (c, *nos-GAL4,UAS-N-cad^{RNAi};miR-9a[E39]*, n=16) immunostaind for N-cad (red) and Vasa (green**)**. Note overall reduction in N-Cad expression when *N-cad^{RNAi}* is driven in GSCs. Scale-bars, 10 µm.

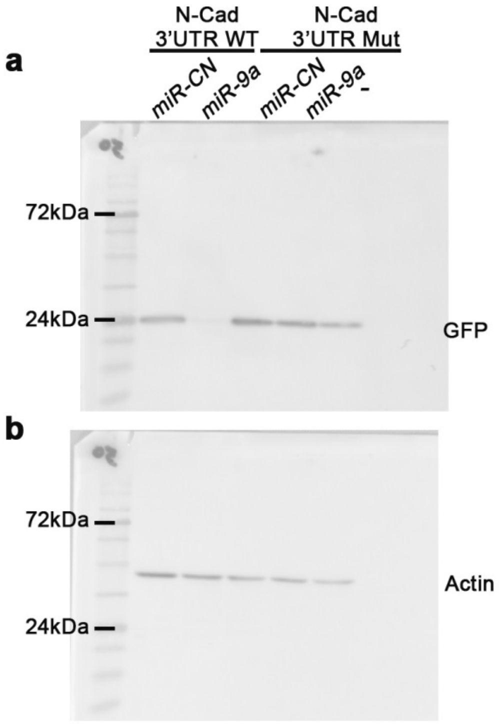

Supplementary Figure 4: ***miR-9a* downregulates GFP-*N-cad*-3'UTR reporter via two recognition sites.** Experimental design is described in legend to Fig. 3e. This uncropped blot displays levels of GFP protein **(a)** and actin **(b).**
